# Changing epidemiology of *Salmonella* Enteritidis human infections in the Netherlands and Belgium, 2006 to 2019: a registry-based population study

**DOI:** 10.2807/1560-7917.ES.2022.27.38.2101174

**Published:** 2022-09-22

**Authors:** Linda Chanamé Pinedo, Eelco Franz, Maaike van den Beld, Nina Van Goethem, Wesley Mattheus, Kees Veldman, Thijs Bosch, Lapo Mughini-Gras, Roan Pijnacker

**Affiliations:** 1 Centre for Infectious Disease Control, National Institute for Public Health and the Environment (RIVM), Bilthoven, the Netherlands; 2 Institute for Risk Assessment Sciences, Utrecht University, Utrecht, the Netherlands; 3 Epidemiology and public health, Sciensano, Brussels, Belgium; 4 National Reference Centre for Salmonella and Shigella, Sciensano, Brussels, Belgium; 5 Wageningen Bioveterinary Research (WBVR), Lelystad, the Netherlands

**Keywords:** Trends, outbreaks, invasive infections, surveillance, restricted cubic spline

## Abstract

**Background:**

Salmonellosis remains the second most common zoonosis in the European Union despite a long-term decreasing trend. However, this trend has been reported to have stagnated in recent years, particularly for *Salmonella enterica* serotype Enteritidis (SE).

**Aim:**

To describe temporal changes in the incidence of SE human infections, and in its associated factors between 2006 and 2019. In addition, we aim to determine which factors influenced the stagnated trend seen in recent years.

**Methods:**

Data on culture-confirmed SE human infections from national surveillance registries in the Netherlands and Belgium between 2006 and 2019 were analysed using multivariable negative-binomial regression models with restricted cubic splines.

**Results:**

SE incidence was significantly higher in summer and autumn than winter, in persons aged 0–4 years and 5–14 years than in persons ≥ 60 years, and increased with increasing proportions of travel-related and resistant SE infections. SE incidence decreased significantly in both countries until 2015, followed by an increasing trend, which was particularly pronounced in the Netherlands. Potential SE outbreaks in both countries and invasive infections in the Netherlands also increased after 2015.

**Conclusion:**

The increase in potential outbreaks and invasive infections since 2015 may partially explain the observed reversal of the decreasing trend. While these results provide insights into the possible causes of this trend reversal, attention should also be given to factors known to influence SE epidemiology at primary (animal) production and pathogen genomic levels.

**Public Health impact of this article**

**What did you want to address in this study?**

Salmonellosis is an important zoonosis and one of the leading causes of food-borne disease outbreaks in Europe. The serotype *Salmonella* Enteritidis (SE) causes ca 30% and ca 20% of all human salmonellosis cases in Belgium and the Netherlands, respectively. We were interested in factors that might have influenced the change in the previously long-term declining trend in human SE infections in the two countries in recent years.

**What have we learnt from this study?**

Potential outbreaks in both the Netherlands and Belgium, and severe infections in the Netherlands increased after 2015, which may partially explain the observed change in the trend. However, other factors not captured by this study, may also have played a role such as factors at the primary (animal) production and pathogen genomic level.

**What are the implications of your findings for public health?**

To (re)establish the decreasing trend in human SE infections in the two countries, alongside preventing outbreaks and invasive disease, further investigation into what other factors are at play is necessary.

## Introduction

Salmonellosis continues to be the second most commonly reported zoonosis in humans and it is also one of the leading causes of food-borne disease outbreaks in the European Union (EU) [1]. Although human *Salmonella* infections usually cause self-limiting mild diarrheal symptoms [2], they can sometimes progress outside of the gut and become invasive and life-threatening, requiring hospitalisation and parental antibiotic treatment [3]. In 2019, human *Salmonella* infections caused > 9,000 illnesses and > 1,000 hospitalisations in the EU [1].

*Salmonella enterica* serotype Enteritidis (SE) is responsible for more than half of all human *Salmonella* infections in the EU [1]. In the Netherlands and Belgium, SE is one of the most common serotypes reported, accounting for ca 30% and ca 20% of all human salmonellosis cases in recent years, respectively [4,5]. This serotype is strongly associated with livestock animals, particularly laying hens [6]. Transmission to humans occurs through consumption of a variety of food products, direct contact with animals, and to a limited extent through the environment and via person-to-person [2,7,8].

The incidence of SE human infection significantly declined from 14.6 per 100,000 inhabitants in 2008 [9] to 7.4 per 100,000 inhabitants in 2012 in the EU [10], mainly due to the EU-harmonised *Salmonella* control programmes in poultry [11]. However, the significant decreasing trend stabilised in 2012 [11] and has remained unchanged since [1]. Factors explaining this stagnating trend in SE human infections are yet to be identified and could be categorised into three main levels: the primary (animal) production level, such as possible deficiencies in the existing control measures in laying hen flocks [12]; the public health (population) level, such as changes in SE occurrence/exposure patterns, risk groups, and surveillance infrastructure [13,14]; and/or the pathogen level, such as increased pathogenicity of certain SE strains [15].

We aim to investigate factors at the population level in two neighbouring countries, the Netherlands and Belgium, that could help explain the stagnating SE trend, and possibly reveal better options for control to (re)establish the decreasing trend in SE human infections. More specifically, we aim to: (i) describe temporal trends in the incidence of SE human infection in the Netherlands and Belgium from 2006 to 2019; (ii) identify factors associated with SE incidence (i.e. age and sex distribution, occurrence of outbreaks, invasive infections, travel-related infections, and antimicrobial resistance); and (iii) assess temporal changes in these associated factors. We hypothesise that changes in the aforementioned factors potentially associated with SE incidence may help to explain the hitherto unknown reasons behind the stagnating trend in human SE infections in the Netherlands and Belgium.

## Methods

This was a registry-based population study using national laboratory surveillance data for *Salmonella*.

### Setting and data collection

#### The Netherlands

Notification of non-typhoid salmonellosis is not mandatory in the Netherlands. However, the Dutch surveillance system has been implemented since 1987, and is based on a network of diagnostic laboratories that send *Salmonella* isolates voluntarily with a minimal set of metadata to the National Institute for Public Health and the Environment (RIVM) for antimicrobial susceptibility, and serotyping and subtyping [16-18], without any subselection.

The network of regional diagnostic laboratories consists of laboratories from hospitals as well as from general practitioners and are spread out in all regions in the country. For this study, data from 30 unique laboratories that submitted their *Salmonella* isolates during 2006–19 were used. During the study period, all human *Salmonella* isolates were sent as pure cultures to the RIVM and were sub-cultured on sheep blood agar and checked for purity. From 2006 to 2012, they were serotyped using classical agglutination. From 2013 to 2019, serotyping was performed using a pre-screening with Luminex technique (Xmap Salmonella Serotyping Assay) followed by confirmation with classical agglutination, i.e.: biochemical tests from 2006 to 2014, and from 2015 onwards with Matrix-Assisted Laser Desorption/Ionisation-Time Of Flight (MALDI-TOF) when necessary. The surveillance system covers ca 62% of the Dutch population.

Antimicrobial resistance (AMR) profiling has been performed since 2008 on a random sample of isolates based on an algorithm that selects isolates according to source, sending laboratory, and serotype [16]. For the study period, broth microdilution was employed to obtain the minimum inhibitory concentration (MIC). The European Committee on Antimicrobial Susceptibility Testing (EUCAST) epidemiological cut-offs (ECOFFs) were applied to interpret the MICs as resistant/susceptible. Additional patient data include sex, age, sampling date, residence location, specimen type (faeces, blood, urine, etc.), and travel history if available. Population size per year, age, and sex was obtained from Statistics Netherlands [19].

#### Belgium

The official national reports for human salmonellosis are based on the number of *Salmonella* isolates from cases that are voluntary sent to the National Reference Center (NRC) by peripheral laboratories for confirmation, antimicrobial susceptibility, and serotyping and subtyping [20], without any subselection.

Clinical diagnostic laboratories serve both general practitioners and hospitals which are located in all regions in the country. For the period 2007–12, 161 unique peripheral laboratories reported *Salmonella* data to NRC, whereas for the period 2013–19, 169 unique laboratories reported data to the NRC. All human *Salmonella* isolates received by the NRC during the study period were first cultured on selective XLD medium and further identified using biochemical tests (fermentation of glucose, negative urease reaction, lysine decarboxylase, negative indole test, H2S production, and fermentation of dulcitol). From 2006 to 2018, isolates were serotype by slide agglutination with commercial antisera, according to the Kauffmann-White scheme. Since 2019, identification of the serotype switched to geno-serotyping based on Luminex xTAG and, in case of doubt, identification was done with (MALDI-TOF). The coverage of the NRC surveillance system was estimated to be 85% in 2016–20, based on the results of a laboratory survey and on a capture–recapture study [20].

AMR profiling has been performed on a random subset of the six most prevalent serotypes of *Salmonella* isolates. For the period 2006–16, disk diffusion was employed and the inhibition diameters were obtained. For the period 2017–19, broth microdilution was used to obtain the MICs. The EUCAST’s ECOFFs were applied to interpret the inhibition diameters and/or MICs as resistant/susceptible. Additional patient data include age, sex, sampling date, postal code, clinical data, and recent travel history. Population size per year, age, and sex was obtained from Statbel, the Belgian statistical office [21].

#### Study population and case definition

The study population consisted of patients with a culture-confirmed *S. enterica* infection caused by the serotype Enteritidis in the Netherlands and Belgium during 2006–19. A person could meet this definition more than once if a subsequent SE infection was reported > 3 months apart.

#### Variables

Age was categorised as 0–4, 5–14, 15–59 or ≥ 60 years old, and sex as female or male. Season was categorised as winter (December to February), spring (March to May), summer (June to August), and autumn (September to November). SE infections with isolates from faeces, urine, vomit, sputum, skin, soft tissue abscesses, and wounds were considered as non-invasive, whereas SE infections with isolates cultured from blood, cerebrospinal fluid, peritoneal fluid, pleural fluid, synovial fluid, bone, or other normally sterile sites were considered as invasive [4].

The proportion of SE invasive infections was calculated as the number of SE invasive infections divided by the total number of SE infections of known type (i.e. where the isolates were isolated from). The proportion of SE infections with travel history was defined as the number of SE infection cases with known international travel history divided by the total number of SE infections. The proportion of AMR in the SE isolates was calculated as the number of cases caused by SE isolates resistant to at least one antimicrobial divided by the total number of SE cases with AMR profiling available. Only those antimicrobials that were tested in both countries during the study period and for which the EUCAST’s ECOFFs are available for disk diffusion and MIC for *Salmonella* spp. were selected. This resulted in the selection of six antimicrobials: chloramphenicol, ciprofloxacin, cefotaxime, gentamicin, tetracyclines, and trimethoprim.

### Statistical methods

A visual assessment of the long-term trends and seasonality in the monthly number of SE cases was performed for both countries during the study period. Monthly numbers of SE cases are presented here as median and inter quartile range (IQR), and the other above-mentioned variables as counts with percentages

The crude monthly incidence rate of SE cases over the study period was estimated using an univariable negative-binomial regression model for each country separately. This model consisted of the monthly number of SE cases (response variable) with the annual total population size as an offset term. Linearity in the long-term trend of the incidence was assessed using the Wald test with two degrees of freedom. If the long-term trend was significantly nonlinear, a restricted cubic spline function of years was used with four knots chosen according to Harrell's recommended percentiles (2006, 2010, 2015, and 2019) using the mkspline2 command in Stata (version 17.0, StataCorp, College Station, US) [22]. The crude monthly incidence rate of SE infections was plotted and expressed as incidence rate (IR) per 100,000 inhabitants.

To assess changes in monthly SE incidence as a function of several variables during the study period, a multivariable negative binomial model was fitted for each country. For the Netherlands, the model included year, season, age group, sex, invasive infection, travel history, and AMR, with year-, age group- and sex-specific population size as an offset term. For Belgium, the same variables, apart from travel history, were used. Data on travel history were not available before 2013 and it was therefore excluded from the model.

To account for potential outbreaks leading to extreme monthly incidence values, the monthly proportion of excess SE cases was also included in the model. This proportion was estimated in a separate analysis as the difference between observed and expected SE cases divided by the observed SE cases. The expected number of cases per month were predicted using negative binomial regression analysis. This model consisted of the monthly number of SE cases as the outcome, with the intercept and yearly population size as the offset term. To control for autocorrelation on the outcome variable, a lag effect of one month was added (*Y_t − 1_*). All independent variables were included in the final model regardless of their significance. Linearity in the long-term trend of the incidence was assessed in the same manner as in the univariable model. To plot significant changes in the incidence of SE over time, adjusted incidence rate ratios (IRRs) with respect to the annual long-term trend were displayed from the multivariable negative binomial model for each country.

Significant temporal changes in SE case demographics, the occurrence of potential outbreaks, invasive infections, travel history and AMR were assessed between two time periods identified based on significant inflection points in the temporal trends of each country from the multivariable model. For both countries, this turning point was in 2015, resulting in the time periods 2006–2014 and 2015–2019. Here, the same covariates were considered in both countries, except for travel history in Belgium. To test for a significant change in one of the covariates between the two time periods, an interaction term of each covariate with the two time periods was added to the model. If the interaction term was significant (p ≤ 0.05), it meant a significant change between these two time periods. All reference categories were chosen based on the lowest incidence of SE human infections to show the groups with higher incidence of SE human infection.

A cluster-robust (sandwich) variance estimator was applied to each model to account for multiple infections in the same person. For each variable, IRRs and their corresponding 95% confidence intervals (CI) and p values were calculated. A complete case analysis based on age and sex was performed to accommodate for the number of inhabitants. All analyses were performed using Stata.

## Results

In total, 5,377 SE cases from 2006 to 2019 were reported in the Netherlands, of which 188 cases (3.5%) were excluded because of missing data on age and sex. In Belgium, a total of 8,819 SE cases were reported, with 541 cases (6.1%) being excluded because of missing data on age and sex.

**Figure 1:**
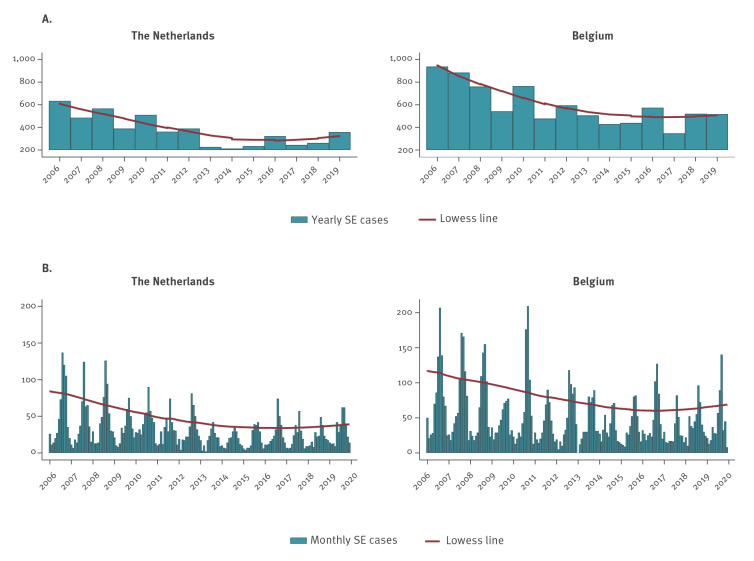
(A) Yearly and (B) monthly number of *Salmonella* Enteritidis (SE) human infection in the Netherlands and Belgium, 2006–2019

Table 1 shows the summary statistics of the study population. Figure 1 displays yearly and monthly SE infections for both countries during 2006–19. Additional summary statistics of the seasonality of SE infections for both countries are provided in Supplementary Figures S1 and S2. The monthly median number of SE cases was 23.5 and 32.0 in the Netherlands and Belgium, respectively. The number of cases was highest in summer in both countries. In the Netherlands, most cases were aged 15–59 years (51%), whereas, in Belgium, the age groups 0–4 years and 15–59 years had most cases (31%). SE cases were similarly distributed between sexes in both countries. The proportion of cases with invasive infection was similar in the Netherlands and Belgium (4% and 5%, respectively). In the Netherlands, the proportion of reported SE cases with known travel history was 17% and the resistance percentage to at least one antimicrobial was 17%. In Belgium, the proportion of reported SE cases with known travel history was 5% (since 2013) and the resistance percentage to at least one antimicrobial was 19%.

**Table 1:** Summary statistics of human *Salmonella* Enteritidis human infection in the Netherlands and Belgium, 2006–2019

Variables	The Netherlands		Belgium	
Monthly SE human infections, median (IQR)	23.5 (13–39)		32 (23–70)	
	n	%	n	%
Season				
Winter	566	10.9	879	10.6
Spring	768	14.8	1,190	14.4
Summer	2,115	40.8	3,109	37.6
Autumn	1,740	33.5	3,100	37.4
Age group (years)				
0–4	727	14.0	2,539	30.7
5–14	932	18.0	2,089	25.2
15–59	2,659	51.2	2,552	30.8
≥ 60	871	16.8	1,098	13.3
Sex				
Female	2,670	51.5	4,130	49.9
Male	2,519	48.5	4,148	50.1
Type of infection				
Invasive	180	3.5	376	4.5
Non-invasive	4,891	94.2	7,285	88.0
Unknown	118	2.3	617	7.5
Travel history				
Known travel	880	17.0	180^a^	5.4^a^
Unknown travel	4,309	83.0	3,325	94.6
Antimicrobial resistance (AMR)				
Resistant SE isolates^b^	732	16.9	463	19.2
Susceptible SE isolates^C^	3,588	83.1	1,948	80.8

^a^ Data available from 2013 onwards in Belgium.

^b^ Resistant SE isolates to at least one of following antimicrobials: chloramphenicol, ciprofloxacin, cefotaxime, gentamicin, tetracyclines, and trimethoprim.

^c^ Susceptible to all aforementioned antimicrobials.

SE: *Salmonella* Enteritidis.

The Lowess line shows the long-term smoothed trend of SE cases.

**Figure 2:**
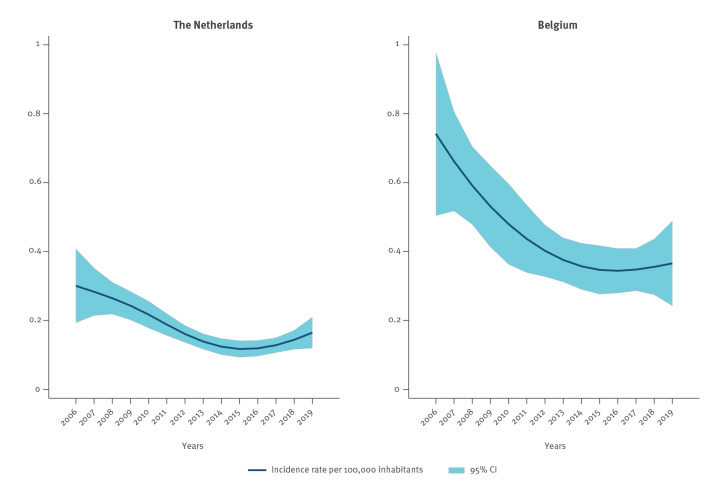
Crude monthly incidence rate of *Salmonella* Enteritidis human infection in the Netherlands and Belgium, 2006–2019

The crude monthly incidence rate of SE cases based on the univariable negative binomial model with restricted cubic splines on the long-term incidence during the study period in both countries is shown in Figure 2. In the Netherlands, the highest monthly IR per 100,000 inhabitants was 0.30 (95% CI: 0.19 – 0.41) in 2006 and the lowest was 0.12 (95% CI: 0.09 – 0.14) in 2015. In Belgium, the highest monthly IR per 100,000 inhabitants was 0.74 (95% CI: 0.50 – 0.98) in 2006 and the lowest was 0.34 (95% CI: 0.28 – 0.41) in 2016.

CI: confidence interval.

Based on the univariable negative binomial model using restricted cubic splines on the long-term incidence with four knots chosen according to the Harrell’s recommended percentiles (2006, 2010, 2015, 2019). In both countries, the SE incidence decreased until 2015, and gradually increased from 2015 to 2019.

### Changes in the incidence of *Salmonella* Enteritidis human infection over time

**Figure 3:**
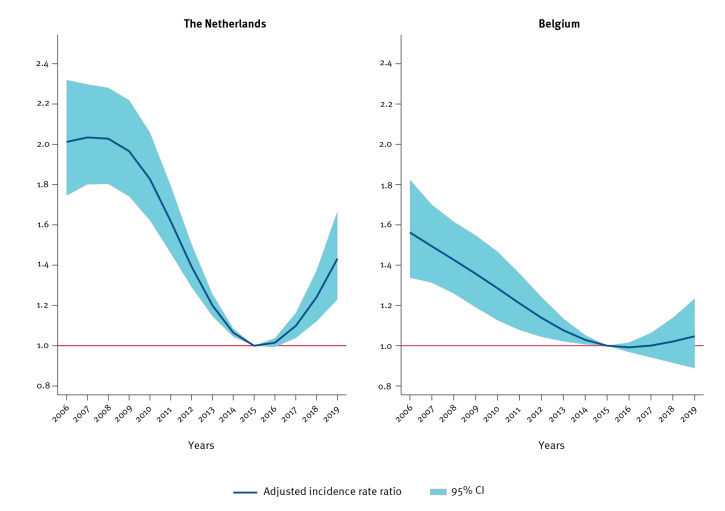
Changes over time in the incidence of *Salmonella* Enteritidis (SE) human infection, with 2006 as reference year, in the Netherlands and Belgium, 2006–2019

In both countries, the incidence of *Salmonella* Enteritidis human infection (SE) cases decreased significantly until 2015, when an upward trend started. While both countries showed a gradual increase in SE incidence from 2015 to 2019, this increase was only statistically significant for the Netherlands (Figure 3).

CI: confidence interval.

Seasonality, autocorrelation, the excess number of SE cases (potential outbreaks), age, sex, invasive infections, travel-related cases, and antimicrobial resistance were accounted for, with the exception of travel history in Belgium due to data only being available from 2013 onwards. The graph is based on the multivariable negative binomial model using restricted cubic splines on the long-term incidence with four knots chosen according to Harrell’s recommended percentiles (2006, 2010, 2015, 2019). The incidence in the Netherlands significantly decreased until 2015, after which it significantly and gradually increased. In Belgium, although the incidence significantly decreased until 2015, the trend increased after 2015. However, the latter trend was not statistically significant.

### Factors associated with *Salmonella* Enteritidis human infection incidence

The results of the multivariable analysis are shown in Table 2. In the Netherlands, the highest incidence of *Salmonella* Enteritidis human infection (SE) was reported in summer (IRR: 3.41, 95% CI: 3.03–3.83), followed by autumn (IRR:2.52, 95% CI: 2.22–2.86), compared with winter. The youngest age group (0–4 years) had a significantly higher SE incidence (IRR: 3.66, 95% CI: 3.27–4.11), as did those aged 5–14 years (IRR: 2.18, 95% CI: 1.93–2.45), and those aged ≥ 60 years (IRR: 1.13, 95% CI: 1.01 – 1.47) than those aged 5–59 years. SE incidence increased with increasing proportions of travel-related and resistant infections (IRR: 1.43, 95% CI: 1.20–1.71 and IRR: 1.29, 95% CI: 1.09–1.52, respectively). No significant association between SE incidence and sex and the proportion of SE infection with invasive infections were found.

**Table 2:** Multivariable negative binomial model of the factors associated with the incidence of *Salmonella* Enteritidis (SE) human infection with restricted cubic splines on the long-term incidence in the Netherlands and Belgium, 2006–2019

Variables	The Netherlands			Belgium		
	Adjusted IRR	95% CI	p value	Adjusted IRR	95% CI	p value
Lag1	1.02	1.02–1.03	< 0.001	1.04	1.03–1.05	< 0.001
Years						
Knot1 (2006 – 2010)	1.05	1.00–1.10	0.072	1.08	1.01–1.15	0.019
Knot2 (2010 – 2015)	0.77	0.66–0.91	0.001	0.81	0.68–0.97	0.019
Knot3 (2015 – 2019)	2.08	1.39–3.12	< 0.001	1.60	1.04–1.05	0.033
Season						
Winter	Reference			Reference		
Spring	1.44	1.27–1.63	< 0.001	1.40	1.22–1.60	< 0.001
Summer	3.41	3.03–3.83	< 0.001	2.94	2.59–3.33	< 0.001
Autumn	2.52	2.22–2.86	< 0.001	2.27	1.97–2.61	< 0.001
Age group (years)						
0–4	3.66	3.27–4.11	< 0.001	9.65	8.79–10.59	< 0.001
5–14	2.18	1.93–2.45	< 0.001	4.61	4.13–5.14	< 0.001
15–59	Reference			Reference		
≥ 60	1.13	1.01–1.26	0.036	1.29	1.13–1.47	< 0.001
Sex						
Female	Reference			Reference		
Male	0.96	0.89–1.04	0.305	1.02	0.94–1.10	0.613
Other variables						
Proportion of excess number of SE cases	1.02	1.02–1.02	< 0.001	1.02	1.02–1.02	< 0.001
Proportion of invasive infection	1.27	0.97–1.66	0.081	1.33	0.98–1.81	0.066
Proportion of travel history^a^	1.43	1.20–1.71	< 0.001	NA		
Proportion of antimicrobial resistance	1.29	1.09–1.52	0.002	1.22	1.07–1.39	0.002

^a^ Not included for Belgium since data were available from 2013 onwards.

In Belgium, the highest incidence of SE was observed in the summer (IRR: 2.94, 95% CI: 2.59–3.33), followed by autumn (IRR: 2.27, 95% CI: 1.97–2.61), compared with winter. The youngest age group (0–4 years) had a significantly higher SE incidence (IRR: 9.65, 95% CI: 8.79–10.59), as did those aged 5–14 years (IRR: 4.61, 95% CI: 4.13–5.14), and those aged ≥ 60 years (IRR: 1.29, 95% CI: 1.13–1.47) than those aged 15–59 years. SE incidence increased with increasing proportions of resistant infections (IRR: 1.22, 95% CI: 1.07–1.39). No significant associations were found between SE incidence and sex and the proportion of SE infection with invasive infections were found.

### Changes in factors associated with *Salmonella* Enteritidis human infection incidence over time

Variables that were associated with changes in the SE incidence between the two study periods (2006–14 vs 2015–19) are shown in Table 3. In the Netherlands, the proportion of the excess number of cases (i.e. potential outbreaks) was significantly higher in 2015–19 than in 2006–14 (p value = 0.001), and was associated with a 1.02 increase in the SE incidence in 2006–14 and a 1.03 increase in 2015–2019. The proportion of SE infection with invasive infection was significantly higher in 2015–19 (p value = 0.032) than in 2006–14, and was associated with 1.04 increase in the SE incidence in 2006–14 and 1.75 increase in 2015–19. There were no significant changes in the age group, sex, the proportion of SE infection with known travel history or AMR. In Belgium, there were significant changes in age group distribution (p value < 0.001). The monthly incidence of SE infection in the youngest two age groups (0–4 and 5–14 years) were a 11.82 and 5.14 increase, respectively, in 2006–14, and a 7.52 and 4.06 increase, respectively, in 2015–19, compared with the 15–59 year age group. The proportion of excess number of SE cases (i.e. potential outbreaks) was significantly higher in 2015–19 than in 2006–14 (p value = 0.002), and was associated with a 1.01 increase in the SE incidence in 2006–14 and a 1.03 increase in 2015–19. There were no significant changes in sex distribution, and the proportion of SE infection with invasive infection and AMR.

**Table 3:** Multivariable negative binomial model with an interaction term between factors associated with the incidence of *Salmonella* Enteritidis (SE) human infection and the two time periods (2006–2014 vs 2015–2019) in the Netherlands and Belgium

Variables	The Netherlands^a^					Belgium ^b^				
	2006–2014		2015–2019		p value^c^	2006–2014		2015–2019		p value^c^
	Adjusted IRR	95% CI	Adjusted IRR	95% CI		Adjusted IRR	95% CI	Adjusted IRR	95% CI	
Age group (years)										
0–4	4.00	3.50–4.57^e^	3.19	2.62–3.90^e^	0.064	11.82	10.52–13.27^e^	7.52	6.49–8.71^e^	< 0.001
5–14	2.38	2.08–2.72^e^	1.88	1.51–2.35^e^		5.14	4.44–5.95^e^	4.06	3.47 – 4.76^e^	
15–59	Reference		Reference			Reference		Reference		
≥ 60	1.22	1.07–1.40^f^	1.00	0.85–1.18^h^		1.55	1.30–1.84^e^	0.98	0.83–1.17^h^	
Sex										
Female	Reference		Reference		0.086	Reference		Reference		0.897
Male	1.01	0.92–1.10^h^	0.87	0.76–1.00^h^		1.03	0.93–1.14)^h^	1.03	0.91–1.16^h^	
Other variables										
Proportion of excess number of SE cases	1.02	1.01–1.02^e^	1.03	1.03–1.04^e^	0.001	1.01	1.01–1.02^e^	1.03	1.02–1.03^e^	0.002
Proportion of invasive infection	1.04	0.74–1.46^n^	1.75	1.21–2.53^f^	0.032	1.20	0.77–1.87^h^	1.75	1.20–2.56^f^	0.204
Proportion of travel history ^d^	1.40	1.13–1.74^f^	1.48	1.08–2.01^g^	0.778	NA		NA		NA
Proportion of antimicrobial resistance (AMR)	1.28	1.04–1.59^g^	1.34	1.04–1.73^g^	0.798	1.33	1.14–1.56^e^	1.13	0.83–1.12^h^	0.188

^a^ Adjusted by years, season, autocorrelation effects, proportion of excess number of SE cases, age group, sex, invasive infections, travel history, and AMR.

^b^ Adjusted by years, season, autocorrelation effects, proportion of excess number of SE cases, age group, sex, invasive infections, and AMR.

^c^ p value of the interaction term

^d^ Not possible to compare between the two time periods in Belgium. Data available only from 2013 onwards.

^e^ p < 0.001.

^f^ p: 0.001–0.01.

^g^ p: 0.02–0.05.

^h^ p > 0.05.

## Discussion

Salmonellosis is the second most commonly reported zoonosis in the EU, after campylobacteriosis, with SE being the most prevalent serotype [23]. Hence, exploring the reasons behind the stagnating trend of SE incidence is important to identify opportunities for its reversal of such a trend. Our study aimed at identifying factors that could potentially explain the non-declining trend in SE human infections in recent years in two neighbouring European countries. To our knowledge, this is the first study assessing which factors might influence the stagnated trend in recent years in Europe.

We observed that, despite the implementation of the EU-harmonised *Salmonella* control programmes and the several years of significant decrease, SE incidence has not been decreasing in both the Netherlands and Belgium since 2015. Indeed, in both countries, a gradual increase in SE incidence was observed from 2015 to 2019, although this increase was statistically significant only in the Netherlands. This was still the case after accounting for population size, age/sex distribution, seasonality, year, the occurrence of potential outbreaks, travel-related cases, invasive infections, and AMR, suggesting that these factors may play a role in the reversal of the decreasing trend, but they cannot fully explain it.

As seen in previous studies in Europe [14,24-26], a higher SE incidence was significantly associated with season, particularly summer and autumn, as well as younger individuals, mostly persons aged 0 to 15 years, travel-related, and AMR in our multivariable negative binomial model, although travel history was not analysed for Belgium due to incomplete data before 2013. It is known that SE infections are more often contracted abroad as compared with the other major serotypes, such as Typhimurium [26,27], which would influence the higher incidence in AMR levels [26,28]. In addition, the fraction of the excess number of monthly SE cases, our surrogate for the occurrence of potential outbreaks, was significantly associated with higher SE incidence, especially during the summer and autumn when SE cases were highly reported in both countries.

There were significant differences between 2006–14 and 2015–19, the turning point of SE incidence, regarding age, potential outbreaks, and invasiveness of infection. In Belgium, there were higher SE infections in the age groups 5–14 and 15–59 in 2015–19 compared with 2006–14 but this was not the case for the Netherlands where the age groups remained unchanged.

The excess numbers of SE cases (i.e. potential outbreaks) and invasive infections had significantly increased in the period of 2015–19 as compared with 2006–14, which in turn may partially explain the stabilising/increasing trend in SE incidence in both countries – although invasive infections did not significantly change during the two study periods in Belgium. Indeed, recent years were characterised by some significant outbreaks of SE in Europe. One of the largest international SE outbreaks mainly linked to eggs from Poland [29] was reported in 2016, which heavily affected the Netherlands and Belgium. Additionally, two more SE outbreaks occurred in 2019: one related to eggs from Spain which was reported in both countries and the other linked to Lahmacum which was only reported in the Netherlands [30,31]. However, the effect of these two outbreaks on the excess number of SE cases was likely smaller due to the lower case numbers. Before 2016, there were no large outbreaks of SE recorded in the Netherlands and Belgium. This is likely because the number of identified SE outbreaks in both countries is heavily skewed towards the era when whole-genome sequencing (WGS) was introduced. In the Netherlands and Belgium, this has been performed for suspected SE outbreaks since 2016. WGS allowed us to identify clusters (e.g. outbreaks) among the bulk of SE cases that would otherwise have been missed. However, such improvements in diagnostic methods used in outbreak investigations did not play a role in the increased number of excess cases (i.e. potential outbreaks) after 2015, as our proxy for potential outbreaks was based on the excess number of SE cases expected for a month in a specific year. Hence, our proxy for potential outbreaks is independent of the diagnostic methods used. Of note, our proxy for potential outbreaks might not only have identified outbreaks, but could be related to other reasons for an increase in the number of SE cases above the baseline, such as increased travel or even natural fluctuations in the number of SE cases. The significant increase in invasive infections in 2015-19 in the Netherlands is in agreement with a recent study that showed a significant increase in invasive non-typhoidal human salmonellosis in 2015-2018 in the Netherlands [4]. Here, it was hypothesised that this could be due to increased virulence of Typhimurium and Enteritidis strains.

In this study, we focused on factors associated with SE incidence at the population level. Future research should focus on factors other than those studied here, such as urbanisation degree and socioeconomic status, which may help in identifying differences in the acquisition of SE human infection. Moreover, a next step would be to explore factors not only at the population level but also at the primary animal production and/or SE strain pathogenicity levels that could potentially play a role in the observed trends. These factors would indeed entail a higher exposure to potential (and possibly hitherto under-recognised) sources of infection and/or the emergence of new strains capable of causing more invasive infections in recent years [15]. In addition, due to the recent coronavirus disease (COVID-19) pandemic and control measures taken worldwide, such as reduced travel, fewer restaurant visits, changes in eating habits, better hand hygiene, etc., it is important to evaluate the impact of such measures on SE incidence after 2019.

This study has some limitations. It was not possible to study the effect of travel history on the SE incidence in Belgium. However, since the effect of travel on SE incidence between both time periods was negligible for the Netherlands, it is unlikely to be an important factor in Belgium. In addition, we cannot completely rule out reporting bias in travel history and type of specimen – the latter used as a proxy of invasive infection in this study – in the surveillance data in both countries, as it is unknown whether this reporting bias has been constant over the study period, resulting in the underestimation of these two factors in the incidence of SE. Additionally, the 62% coverage for *Salmonella* human infections in the Netherlands could potentially affect generalisability of our findings in this country. Finally, surveillance data usually miss SE human infection cases with mild symptoms who do not seek medical care and therefore are not reported in the surveillance system. This may result in selection bias and underestimation of the SE incidence.

## Conclusions

SE incidence is no longer decreasing in the Netherlands or Belgium. While a statistically significant increase has been seen in the Netherlands from 2015 onwards, Belgium has shown a similar trend, albeit not (yet) statistically significant. Although the situation may change in the coming years, it has been shown that the COVID-19 pandemic has had a significant impact also on this as well as other infectious diseases, thus any further monitoring of SE trends after 2020 will be challenged by the implemented public health measures and altered healthcare-seeking and diagnostic behaviours. Regardless, our study showed that increased SE incidence was associated with season, particularly summer and autumn, younger individuals, travel-related cases, AMR, and the occurrence of potential outbreaks. In particular, the occurrence of potential outbreaks in both countries and invasive infections in the Netherlands has increased after 2015, which might partially explain the observed trend in SE incidence. Although the effect of these factors on SE incidence may vary situationally, they offer opportunities for the identification of targets for intervention and further investigation into possible causes of the stagnating SE trend. Yet, other factors at the primary (animal) production and pathogen genomic levels need to be further elucidated.

## Supplementary Data

269000 Supplementary Material
