# Supplementary material for: Changing epidemiology of Salmonella Enteritidis human infections in the Netherlands and Belgium, 2006 to 2019: a registry-based population study
Source: Euro Surveill. 2022 Sep 22;27(38):2101174. doi: 10.2807/1560-7917.ES.2022.27.38.2101174 (PMC9511682; doi:10.2807/1560-7917.ES.2022.27.38.2101174)

This supplementary material is hosted by Eurosurveillance as supporting information alongside the article “Changing epidemiology of *Salmonella* Enteritidis human infections in the Netherlands and Belgium, 2006 – 2019: A registry-based population study”, on behalf of the authors, who remain responsible for the accuracy and appropriateness of the content. The same standards for ethics, copyright, attributions and permissions as for the article apply. Supplements are not edited by *Eurosurveillance* and the journal is not responsible for the maintenance of any links or email addresses provided therein.

Figure 1 Seasonality of *S.* Enteritidis human cases by month and year in the Netherlands and Belgium, 2006 to 2019.

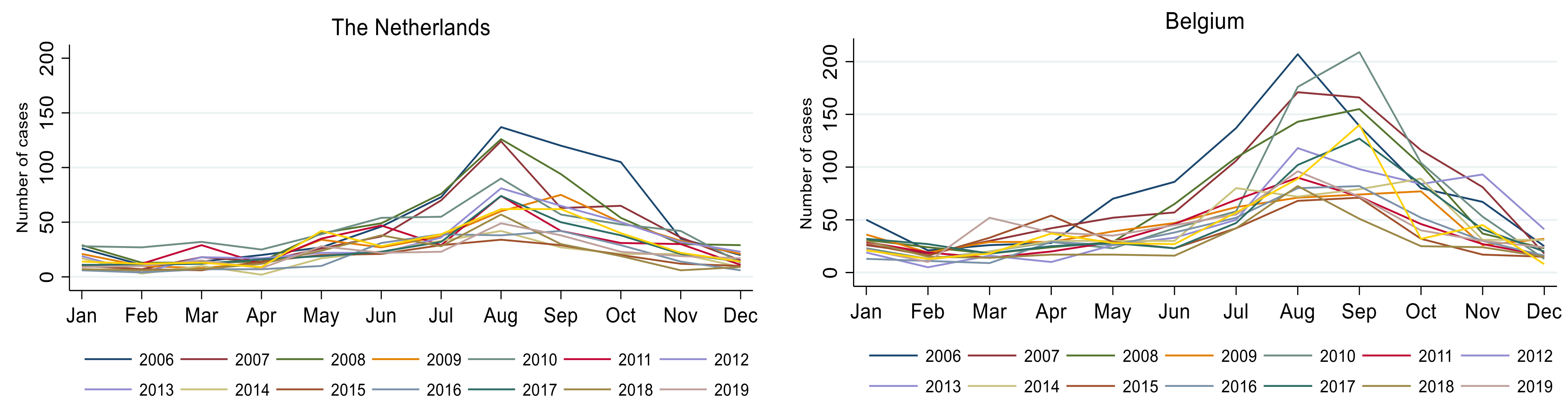

Figure 2. Monthly average, minimum and maximum number of *S.* Enteritidis human cases reported in the Netherlands and Belgium, 2006-2019

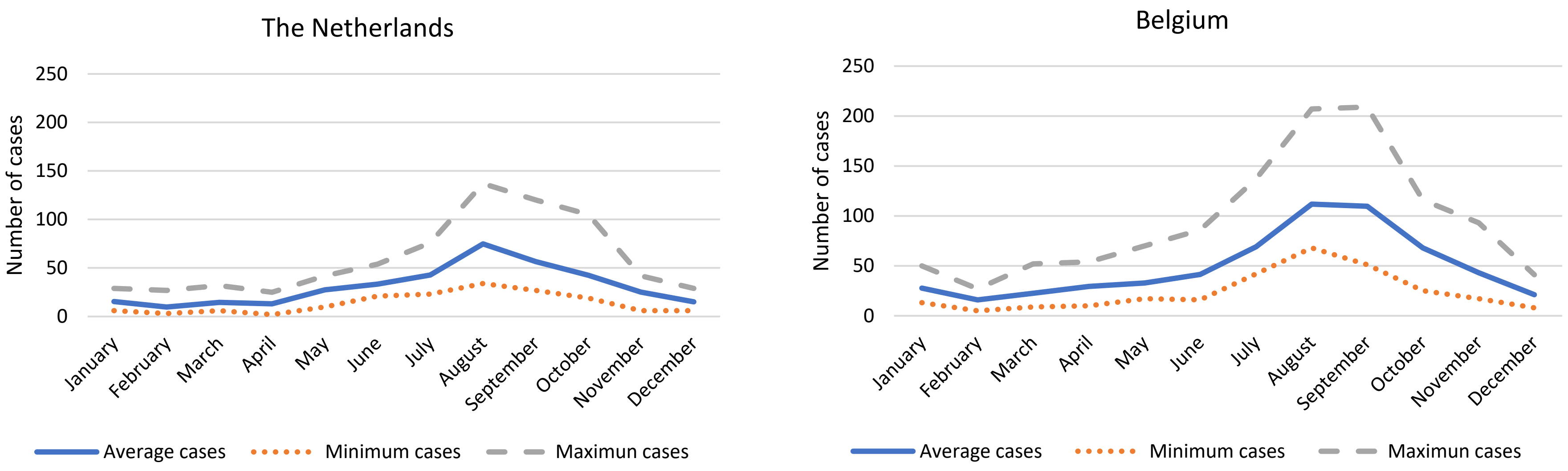

Supplement: Supplementary Material [file 2101174_PINEDO_Supplementary_material.pdf]
